# Study on the Impact of Government Health Expenditure Equity on Residents’ Health Level in the Chengdu–Chongqing Economic Circle of China

**DOI:** 10.3390/ijerph191912758

**Published:** 2022-10-05

**Authors:** Haidong Yu, Yujie Peng, Lanfang Pu

**Affiliations:** 1 School of Public Health, Chongqing Medical University, Chongqing 400016, China; 2 Research Center for Medicine and Social Development, Chongqing Medical University, Chongqing 400016, China; 3 Research Center for Public Health Security, Chongqing Medical University, Chongqing 400016, China

**Keywords:** equity of government health expenditure, residents’ health level, Thiel index, system GMM model

## Abstract

Background: When the global COVID-19 epidemic continues to spread, residents pay more attention to their health. This paper studies the relationship between the equity of government health expenditure and the health level of residents. Methods: The Theil index and a principal component analysis were used to measure the equity of government health expenditure and the health level of residents in the Chengdu–Chongqing economic circle. Then, an empirical study on the relationship between the equity of government health expenditure and the health level of residents in this region was conducted with the System GMM model. Results: 1. The Theil index rose from 0.0115 in 2015 to 0.0231 in 2017 and gradually decreased to 0.0106 in 2020. 2. The overall health level of residents showed an upward trend, rising from 1.95 in 2015 to 2.33 in 2017, then remained high and fluctuated slightly. 3. There was a positive correlation between the Theil index and the health level of residents at a significance level of 1% (*β* = 0.903, *p* < 0.01). Conclusions: 1. The Theil index was close to 0, indicating that the equity of government health expenditure in the Chengdu–Chongqing economic circle was generally good. 2. The health level of residents in the Chengdu–Chongqing economic circle had improved compared to before. 3. The fairer the government’s health expenditure, the higher the residents’ health level.

## 1. Introduction

Under the background of the COVID-19 epidemic and the “Healthy China 2030” strategy, residents’ health level has attracted increasing attention. Improving the health level of residents has become the only way to achieve high-quality development. Therefore, studying the impact of government health expenditure on residents’ health will benefit the region and individuals.

Many factors affect residents’ health levels, and government health expenditure is essential [1,2]. It is closely related to the quality of national medical and health services, the accessibility of national services, and the equity of enjoying medical services [3]. Generally, the equity of government health expenditure is the equality of public access to the opportunities, quantity, and quality of medical and health services provided by health expenditure [4].

The equity of government health expenditure can be examined from the expenditure structure and burden ratio. The expenditure structure includes four parts. Firstly, there are regional differences in government health expenditure [5]. Some research about China [6] showed that the gap was the largest between East and West, the middle between Central and West, and the smallest between East and Central. However, other research [7] argued that although the Chinese government had tilted public health expenditure towards poor provinces in the central and the western regions, the imbalance and inequity in allocating public health resources among provinces are still expanding.

Secondly, government health expenditures are different between urban and rural areas. As a result, there is a massive gap between China’s urban and rural floating populations regarding access to medical services, vaccination coverage, accidents, and injuries [4,8].

Thirdly, there are institutional differences in government health expenditure. China’s medical system implements a hierarchical system, and the hospital level determines the resource allocation [9], leading to the concentration of high-quality medical resources in large hospitals.

Finally, there are medical-technician-related differences in government health expenditure. Large medical institutions “siphon” the primary medical and health resources. Promising talents and funds flow to large medical institutions, while the primary medical institutions are short of talent [10].

The expenditure burden ratio includes two parts: The government health expenditure is increasing yearly, but its burden ratio is not ideal. As a result, the proportion of government health expenditure in total health expenditure is decreasing, while the burden of personal health expenditure is increasing. When low-income groups face a rapid rise in medical expenses, the only choice is to delay minor diseases and resist significant diseases, ultimately losing both human and financial resources [11,12].

Under the background of the epidemic of COVID-19 and the “Healthy China 2030” policy, the government, scholars, and residents attach great importance to health. To improve the health status of residents, the government has consciously adjusted and tilted its policies, and the inequity of its health expenditure is gradually improving [13,14,15,16]. The research [14] showed that after the Chinese government launched the Major Illness Medical Insurance Innovation Plan, the proportion of government health expenditure burden had increased, which would significantly reduce the impact of families’ major illness medical expenditure. Another study [17,18] found that residents’ medical treatment equity had been improved to a certain extent due to the government’s efforts. The gap between different income groups in medical security, hospitalization compensation, and health service utilization has been significantly narrowed, and residents’ health levels have improved.

The research on the impact of residents’ health levels mainly focused on education and income. The *Lancet Public Health* [19] found that education was the leading cause of health inequality. Zajacova and Lawrence [20] found that adults with higher education live healthier and longer than their peers with lower education. Grossman [21] used the concept of health capital to construct a health demand model, studied the relationship between education level and health level, and concluded that education had a significant positive correlation with health level. Previous studies about income had found that low income was the main reason preventing the poor from seeking medical services, and it was also the reason for the deterioration in poor people’s health [22,23]. Most literature proved a significant positive correlation between income and health [24,25,26]. Vanzella and Veenstra [27] found that among the low-income population, the general self-rated health status was poor, and there were long-term diseases or health problems. Among the high-income population, women generally self-rated their health better. However, some studies believed that relative income had a significant negative impact on health [28], and the income gap significantly negatively impacted the health level of rural residents [29].

The previous research mainly focused on the “equity” of government health expenditure and the “influencing factors” of health level. Although it also involved the subjective judgment about the equity of government health expenditure’s impact on Residents’ health level, there lacked corresponding empirical research. How should we measure the equity of government health expenditure? What is the health status of residents? What is the impact of the equity of government health expenditure on the health of residents? All these questions need to be studied carefully from the empirical perspective. Based on this, we took the Chengdu–Chongqing economic circle as the research object. We started with the internal relationship between the equity of health expenditure and the health level of residents; then, the impact relationship between the equity of government health expenditure and the health level of residents was studied using an empirical perspective. Related studies provide a reference for constructing a healthy China and achieving high-quality development. The potential contributions of this paper are as follows: First, the research perspective of government health expenditure was extended. The previously published literature used quantitative analysis and was generally carried out from the scale of government health expenditure. This research was carried out from the perspective of government health expenditure equity. Second, residents’ health level was comprehensively measured by principal component analysis, which is more comprehensive, objective, and persuasive than the mortality rate used by the general literature as a health proxy variable. Third, empirical research was conducted on the relationship between the equity of government health expenditure and residents’ health level.

## 2. Materials and Methods

### 2.1. Research Object

In January 2020, the Central Committee of the Communist Party of China made a significant decision and deployment to promote the construction of the Chengdu–Chongqing economic circle and create a vital growth pole for high-quality development. In October 2021, the outline of the construction plan of the economic circle was issued. As the ultimate economic growth pole in inland China, the Chinese government has high expectations for Chengdu and Chongqing. Therefore, driven by the government’s policies and the newly defined regional scope as the starting point, this paper selects the Chengdu–Chongqing economic circle and its residents as the research object.

### 2.2. Measurement of Government Health Expenditure Equity—Thiel Index

The Theil index [30] was used to measure the equity of government health expenditure. It is an indicator of the degree of inequality between regions. A greater Theil value indicates higher inequity. The index and its decomposition can be measured based on “the perspective of local population distribution” and “the perspective of regional GDP.” In this paper, the first one was used because. compared with GDP distribution in each region, population distribution is generally better. In addition, the spatial balance of the population is also better than that of GDP. In addition, one of our research topics is the health level of residents, which is related to the population. Therefore, it is more reasonable to use the population perspective. The calculation formula of the Thiel index is as follows:

(1) $$\mathrm{Totality}\;\mathrm{Thiel}\;\mathrm{index}:\;T_{Theil}=\sum \frac{y_{m}}{Y}\times \ln\left(\frac{y_{m}/Y}{x_{m}/X}\right)$$

(2) $$\mathrm{Between}\;\mathrm{regional}\;\mathrm{Theil}\;\mathrm{index}:\;T_{BR}=\sum \frac{Y_{i}}{Y}\times \ln\left(\frac{Y_{i}/Y}{X_{i}/X}\right)$$

(3) $$\mathrm{Within}\;\mathrm{regional}\;\mathrm{Theil}\;\mathrm{index}:\;T_{WR}=\sum \frac{Y_{i}}{Y}\times \sum \frac{y_{m}}{Y_{i}}\times \ln\left(\frac{y_{m}/Y_{i}}{x_{m}/X_{i}}\right)$$

(4) $$\mathrm{Totality}\;\mathrm{Thiel}\;\mathrm{index}:\;T_{Theil}=T_{WR}+T_{BR}$$

*x*_m_ and *y*_m_ represent a prefecture-level city’s total population and government health expenditure, respectively. Xi and Yi represent the region’s total population and government health expenditure (Chongqing economic circle or Chengdu Economic Circle), and X and Y represent the total population and government health expenditure of the Chengdu–Chongqing economic circle, respectively. The smaller the Theil index is, the more equitable the government health expenditure is. On the contrary, the larger the Theil index is, the more inequitable the government health expenditure is. This paper used the Theil index to comprehensively and objectively evaluate the equity of government health expenditure in the Chengdu–Chongqing economic circle and provide essential data for subsequent empirical tests.

### 2.3. Measurement of Residents’ Health Level—Principal Component Analysis

This paper used the principal component analysis method [31] to comprehensively measure residents’ health levels. Currently, most of the existing literature adopts proxy indicators of health level, such as self-rated health [27], to evaluate and analyze the health level. However, proxy indicators can only be regarded as one aspect of health performance, and the subjectivity is too strong. The health level of residents is a system that can be comprehensively reflected by residents’ health status, medical conditions, and other indicators. If one of the indicators is used as a proxy indicator, it will lose a lot of useful information. The principal component analysis method used in this paper has the characteristics of dimension reduction. It can capture the primary information from multiple indicators and then form a comprehensive indicator [32,33]. Thus, the health level of residents can be measured well.

Based on the national policy (Healthy China 2030) and the research foundation [34], this paper used principal component analysis to measure and evaluate residents’ health levels and provide primary data for subsequent empirical tests.

### 2.4. Research Hypothesis

**Figure 1:**
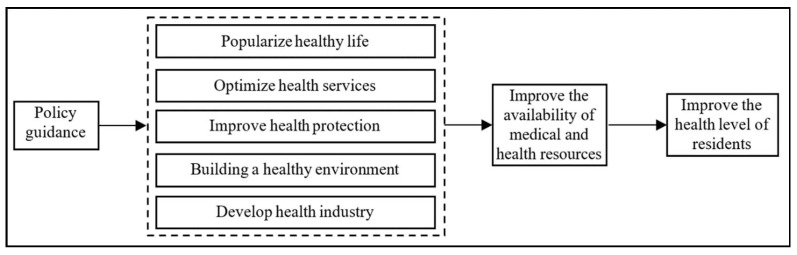
Influence channels of government policies on Residents’ health level.

**Figure 2:**
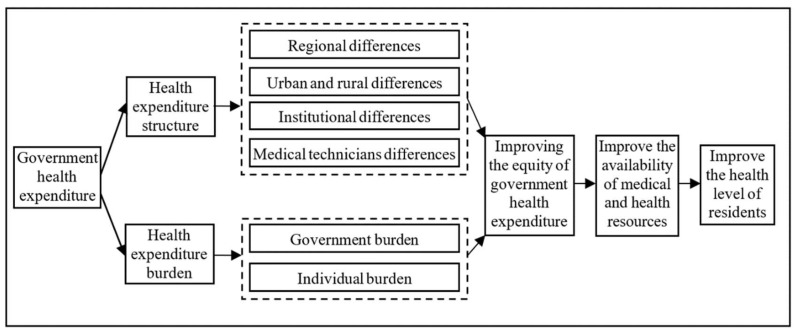
Influence Channels of the government health expenditure equity on residents’ health level.

Based on the introduction part of the article, the difference in government health expenditure is mainly reflected in the expenditure structure and burden. The structural difference in government health expenditure can be divided into regional, urban–rural, institutional, and medical technician differences. The difference in government health expenditure burden can be divided into government burden proportion and individual burden proportion. Based on these differences in government health expenditure, the Chinese government has implemented significant policies, such as popularizing a healthy life, optimizing health services, improving health security, building a healthy environment, and developing the health industry. Under the guidance of the government’s many policies for the benefit of the people, the residents’ access to medical and health resources has been significantly improved. As a result, the residents’ health level has been continuously improved, and the core health indicators of the residents are among the highest in among middle- and high-income countries in the world. From 2010 to 2020, China’s per capita life expectancy increased from 74.83 years to 77.3 years, an increase of 2.47 years. According to the Outline for Women’s Development in China (2011–2020) released by the National Bureau of statistics in 2020, the average life expectancy of Chinese women exceeded 80 years, four years higher than the world average. This paper proposes the following two research hypotheses based on the above analyses (Figure 1 and Figure 2).

**Hypothesis** **1:** Under the guidance of government policies, the health level of residents in the Chengdu–Chongqing economic circle has been improved compared with that before.

**Hypothesis** **2:** There is a positive correlation between the equity of government health expenditure and the health level of residents.

### 2.5. Model Setting and Variable Description

In terms of research methods, the static panel assumes that there is no lag effect in the explained variables. Everything has a continuous development process, and the health level of the previous period will inevitably affect the health level of the next period. Therefore, this paper used the dynamic panel regression model (System GMM model) with mitigation endogeneity for analyses. Health level was used as the explained variable, and Health_i,t-1_ represents a lag term of residents’ health level (Formula (5)). The core explanatory variable is the Theil index, which reflects the equity of government health expenditure. It is a reverse indicator, so the government health expenditure is more inequitable when the index is higher. The measured Theil index was multiplied by the minus 1 in the empirical analysis to convert it into a positive indicator. Other control variables are nominal education expenditure, personal disposable income, residents’ deposits, and each region’s nominal per capita GDP. Table 1 and Table 2 represent the specific setting and descriptive statistics of indicators.

health_i,t_ = β_0_ + β_1_health_i,t-1_ + β_2_theil_i,t_ + β_3_edu_i,t_ + β_4_pdi_i,t_ + β_5_dep_i,t_ + β_6_gdp_i,t_ + Ɛ_i,t_ (5)

## 3. Results

### 3.1. Measurement of the Equity of Government Health Expenditure

**Figure 3:**
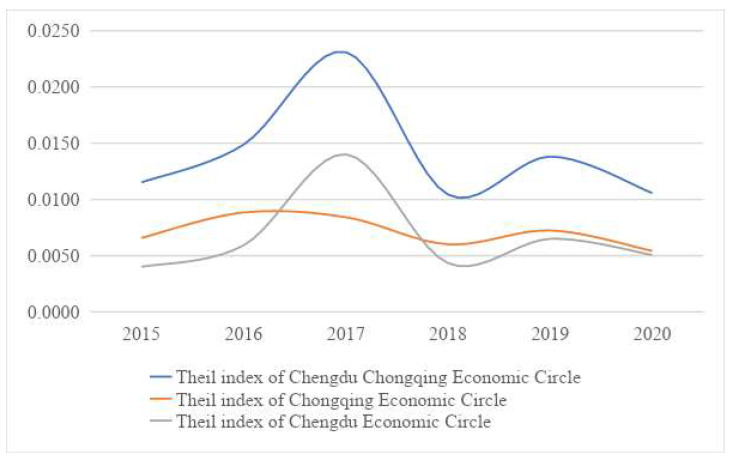
The trend of the Thiel index.

This article used the Theil index and its decomposition method to measure the equity of government health expenditure. The results are shown in Table 3 and Figure 3.

During the study period, the Theil index of the Chengdu–Chongqing Economic Circle showed a trend of rising first and then decreasing. The Theil index increased from 0.0115 in 2015 to 0.0231 in 2017, then decreased to 0.0106 in 2020. The Theil index trend of the Chengdu economic circle was similar to that of the Chengdu–Chongqing economic circle, which also increased first and then decreased. However, that of the Chongqing economic circle was different. From 2015 to 2020, it stayed low, with a slight fluctuation range. The maximum value was 0.0088 in 2016, and the minimum was 0.0054 in 2020, indicating that the government health expenditure in the Chongqing economic circle was very fair. To sum up, as far as the three regions were concerned, their Theil index values were at a low level, indicating the equity of government health expenditure in the three regions was equal, and the difference was negligible (Table 3 and Figure 3).

Another finding was that the Theil index trend of the Chengdu Economic Circle and the Chengdu–Chongqing economic circle was almost the same, with the peaks and troughs basically the same. The main reason is that the government health expenditure and the population in the Chengdu economic circle account for more than two-thirds of the Chengdu–Chongqing economic circle, 72% and 69%, respectively.

In 2017, the Theil index of the Chengdu–Chongqing economic circle and the Chengdu economic circle reached the maximum values, 0.023 and 0.014, respectively (Table 3 and Figure 3), indicating that it was the most inequitable year for the government health expenditure in these two regions. The main reason was that although the government health expenditure and population scale increased in 2017, the growth rates decreased.

Table 4 shows that the contribution of within-regional differences is much higher than that of between-regional differences, indicating that within-regional differences are the main reason for the inequity of public health investment. Furthermore, the within-regional differences tend to expand gradually, with the contribution rate of within-regional differences exceeding 90%.

### 3.2. Measurement of Health Level of Residents

**Figure 4:**
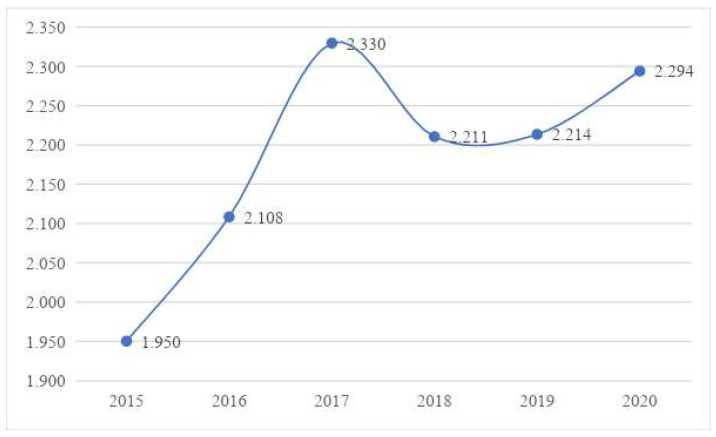
Changes in the trend of residents’ health level.

The measurement results showed that during the study period, the health level of the residents in the Chengdu–Chongqing Economic Circle showed an upward trend, rising from 1.95 in 2015 to 2.33 in 2017, and then remained at a high level and slightly varied between 2017 and 2020. Furthermore, it showed that the local governments in the Chengdu–Chongqing Economic Circle followed the national strategy, constantly reduced the difference in government health expenditure, improved the availability of medical and health resources, and further improved the health level of residents. Therefore, research hypothesis 1 has been verified (Figure 4).

### 3.3. Empirical Research Results and Hypothesis Verification

#### 3.3.1. Analysis of regression results of dynamic panel data

This study used the system GMM method to estimate the model. The test results are shown in Table 5. First, the Hansen test *p* values of models were all above 0.1, which meant that the instrument variables were valid. Second, the *p* values of AR(2) were more significant than 0.1, which indicated that the GMM estimators of the system were consistent, and there was no second-order autocorrelation in the model. Therefore, the model setting was reasonable, and the conclusion was accurate and reliable.

As shown in the last column in Table 5, the stepwise regression results of each explanatory variable had a good consistency. The equitable government health expenditure (Theil) was significantly and positively correlated with the health level of residents (*β* = 0.903, *p* < 0.01), indicating a noticeable promoting effect. The fairer the government health expenditure, the higher the health level of residents. Research hypothesis 2 has been verified. In addition, the lag coefficient of residents’ health levels was positive, with a significant promoting effect (*β* = 0.374, *p* < 0.01), indicating that the change in residents’ health levels was greatly affected by its change in the previous period. Residents’ health levels had strong inertia. The regression results also found that education expenditure (edu) was significantly positively correlated with residents’ health level at the level of 1% (*β =* 0.021, *p* < 0.05). The residents’ deposit (dep) was significantly negatively correlated with the residents’ health level (*β* = −0.034, *p* < 0.01).

#### 3.3.2. Stability Test: Regression Analysis of Static Panel Data Model

To obtain stable and reliable research conclusions, we used other empirical models to test the robustness of the research hypothesis, e.g., the random effect model and the fixed effect model (Table 6). Considering that each region has specific development conditions and there would be missing variables that do not change with time, the fixed effect model was used for empirical tests. On the other hand, the Hausman test result “prob > chi2 = 0.0000” firmly refuted the original hypothesis of the random effect model, so the empirical conclusion of the fixed effect model was more reasonable. In addition, taking the fifth column of the fixed effect model in Table 6 as an example, the “rho = 0.9894” in the individual fixed test results indicated that the variance in the compound interference term mainly comes from the individual effect change.

The random effect model, fixed effect model, and System GMM model were consistent (Table 5 and Table 6). Therefore, the equity of government health expenditure was significantly positively correlated with the health level of residents at the level of 1%, which indicated that the results of this study were stable and reliable.

In addition to considering the individual differences in various regions, the time effect was also considered. The joint significance test result of the time dummy variable “prob > chi2 = 0.0115” strongly rejected the original hypothesis of “no time effect,” i.e., there was a time effect. Therefore, this article adopted a two-way fixed effect model for the robustness test (Table 7).

In Table 7, column 5, the estimated coefficient of the Theil index is 0.462. The 1% significance test showed that the equity of government health expenditure had a positive impact on the health level of residents, i.e., the fairer the health expenditure, the higher the health level of residents. This result was consistent with the results of the System GMM model and further showed that the results of this paper were stable and reliable.

## 4. Discussion

Table 8 reports the regression results of the impact of the equity of government health expenditure on the health level of residents. Column (1), column (2), column (3), and column (4) represent the regression results of the one-step system GMM model, the two-step system GMM model, the individual fixed effect model, and the two-way fixed effect model, respectively.

As revealed in the results of the two-step system GMM model in Table 8, the core explanatory variable health expenditure equity was significantly and positively correlated with the residents’ health level at 1% (Table 8, column 2, *β* = 0.903, *p* < 0.01). The main reasons for this finding are the following:

First, government guidance. As the government’s primary role in promoting residents’ healthy development, the relevant laws and regulations, policy documents, and industrial standards issued by the government have played a leading and guiding role in the whole field of residents’ health. For example, “Healthy China 2030” was the most critical policy guidance document issued by the Chinese government.

Second, system reform. Government functional departments and medical and health institutions are essential participants in the whole health field, so their every move was related to the residents’ health field development. A series of medical security system reforms significantly reduced the burden of medical care, improved people’s livelihood, and maintained social harmony and stability, for example, by improving the equity and appropriate treatment guarantee mechanism, promoting the supply side reform of medical services, establishing an efficient and effective medical insurance payment mechanism, and optimizing the public management service of medical security, which government functional departments continuously promote.

Third, residents’ self-health awareness. With the COVID-19 epidemic still prevalent, the health awareness of the whole population is gradually enhanced [40], and the public is generally accepting healthy eating habits and regular work and rest times. From the above analyses, the equity of government health expenditure in society had improved, and residents’ health levels had improved. At the same time, it also verified research hypothesis 2 of this paper. The equity of government health expenditure had a positive effect on the health level of residents.

Other researchers also reported similar conclusions. Yip W. et al. [17] found that to provide citizens with equal basic medical services, the Chinese government has tripled health expenditure funding and given special help to people with low socio-economic status. China has made significant progress in improving equal access to health care and strengthening economic security. Meng Q et al. [18] evaluated the trend of medical care accessibility and financial security after China’s healthcare system reform. They found that the three indicators of access to coverage, healthcare activities, and financial protection have been greatly improved, indicating that the government can improve the equity of residents’ medical treatment through policy guidance. In the study of Flat ø and Zhang [41], they believed it was essential to improve China’s health equity and realize the equalization of health insurance plans. The universal health coverage reforms should strengthen the support of grassroots medical departments and restrict the unnecessary use of high-level hospitals to promote the equity of medical services.

In Table 8, column 2, education expenditure (edu) was significantly positively correlated with the health level of residents (*β* = 0.021, *p* < 0.01). The increase in government education expenditure can lead to an increase in the number of schools, the enhancement of teachers, and the upgrading of teaching equipment. All these can improve students’ knowledge and cultural level, including their mastery of health knowledge, so their health level will improve. Other studies [19,20,21,42,43] had similar conclusions. They believed that the more education funds were invested, the healthier the residents would be. There was also an obvious positive correlation between education and health. Policies that affect the level of education would have a significant impact on population health. However, there were also studies with opposite results. Mazumder [44] found no causal relationship between education and health. This result may be affected by the authors’ choice of compulsory education law as a variable.

In Table 8, column 1, residents’ deposits had a significantly negative correlation with residents’ health level (*β* = −0.034, *p* < 0.01), i.e., the increase in residents’ deposits would inhibit the health level of residents. This conclusion was similar to Ouyang Shengye’s [45] study, which found a significant negative correlation between residents’ health status and their household savings, i.e., the deterioration of residents’ health status would lead to an increase in household savings. This conclusion seems to be contrary to the general cognition in reality. Actually, the more residents save, the stronger their purchasing power and desire will be. That means residents’ access to medical services would be improved, and the health level of residents would also rise accordingly. However, the empirical conclusion was that the increase in residents’ savings would reduce the health level of residents. When residents deposit their cash in the bank, the funds that can be used for current consumption are delayed for future consumption. The possible reasons are as follows: First, for the residents’ health, since the funds are in the bank and there is no major disease, the residents will not be eager to withdraw money from the bank to see a doctor and will delay seeing a doctor and buying medicine, which means that the residents’ health level will decline. Second, the health of residents themselves is not optimistic. To ensure they have enough funds to see doctors in the future, the residents may deposit more funds in the bank in advance.

## 5. Conclusions

Based on the research hypothesis and empirical tests, this paper draws the following three main conclusions: (1) According to the measured Theil index, the equity of government health expenditure in the Chengdu–Chongqing economic circle had experienced a process from good to bad, and then from bad to good, but the overall equity was close to 0, indicating that the overall equity of government health expenditure in this region was good. (2) The health level of the residents in the Chengdu–Chongqing economic circle has been improved compared to before, which might be related to the government’s guidance, system reform, and residents’ self-health awareness enhancement. (3) This paper used the system GMM model for empirical research and used the fixed effect model, random effect model, and two-way fixed effect model to test the stability of the results. The results show that the equity of government health expenditure had a positive effect on the health level of residents at a significance level of 1%.

Regarding policy recommendations, first, the radiation range of high-quality health resources in Chengdu and Chongqing should be expanded by establishing regional medical centers. Second, the government should be encouraged to give preferential policies and guidance to economically underdeveloped areas in the health expenditure budget. Financial support should be provided for primary medical and health institutions and preference should be given to primary medical and health institutions to meet the basic health needs of most people and improve the equity of health expenditure. Third, the government should ensure equity, actively carry out counterpart support and assistance, and provide high-quality medical and health resources to residents in underdeveloped areas. Fourth, the government should encourage the participation of social capital and accelerate the construction of a diversified medical pattern.

As a particular area planned by the Chinese government in 2021, the Chengdu—Chongqing Economic Circle includes all prefecture-level cities in Chongqing and Sichuan, which are economically developed with good medical and health conditions. As shown by the results, the equity of government health expenditure in the Chengdu–Chongqing Economic Circle was good, and the health level of residents was also improved compared to before, but this did not mean that there were no problems. On the contrary, for example, in the economically underdeveloped areas adjacent to the western part of the Chengdu–Chongqing economic circle, residents’ health levels are still poor, and the distribution of health resources is unfair. The purpose of the Chengdu–Chongqing Economic Circle, established by the government, is to drive the development of economically underdeveloped regions through economically developed regions. Therefore, in economically underdeveloped areas, how the equity of government health expenditure and whether the health level of residents has been improved will become one of our directions for further future studies.

## Figures and Tables

**Table 1:** Indicators of influencing factors on Residents’ health level.

Variable	Definition	Reference	Data Sources
health	The health level of residents	Hsu et al. (2009) [35]	Author’s calculation
theil	Equity of government health expenditure	Woldemichael et al. (2019) [36]	
edu	Nominal education expenditure	The Lancet Public (2020) [19]	Chongqing statistical yearbook/ Sichuan statistical yearbook
pdi	Personal disposable income	Marmot (2002) [37]	
dep	Deposits of residents	Feng, Chen (2021) [38]	
gdp	Nominal per capita GDP	Boyle et al. (2006) [39]	

**Table 2:** Descriptive statistics of variables.

Variable	Obs	Mean	Std. Dev.	Min	Max
health	264	1.14 × 10^−5^	1.766	−1.486	10.642
theil	264	−0.0319	0.377	−0.758	2.879
edu	264	32.0646	41.506	4.293	327.727
pdi	264	3.49334	1.190	1.384	7.502
dep	264	10.9319	19.500	1.137	171.214
gdp	264	6.0122	2.919	2.093	22.959

**Table 3:** The measurement results of the regional Theil index.

Year	Theil Index of Chengdu–Chongqing Economic Circle	Theil Index of Chongqing Economic Circle	Theil Index of Chengdu Economic Circle
2015	0.0115	0.0066	0.0040
2016	0.0149	0.0088	0.0059
2017	0.0231	0.0084	0.0140
2018	0.0104	0.0060	0.0043
2019	0.0138	0.0072	0.0065
2020	0.0106	0.0054	0.0051

**Table 4:** Theil index and its decomposition in the Chengdu–Chongqing economic circle.

Year	Theil Index	Within-Regional Theil Index	Between-Regional Theil Index	Within-Regional Contribution Rate	Between-Regional Contribution Rate
2015	0.0115	0.0106	0.0009	91.97%	8.03%
2016	0.0149	0.0148	0.0001	99.20%	0.80%
2017	0.0231	0.0224	0.0007	97.11%	2.89%
2018	0.0104	0.0103	0.0001	99.20%	0.80%
2019	0.0138	0.0137	0.0001	99.48%	0.52%
2020	0.0106	0.0105	0.0001	99.28%	0.72%

**Table 5:** Regression results of one-step system GMM and two-step system GMM.

Variable	One-Step System GMM					Two-Step System GMM				
	(1)	(2)	(3)	(4)	(5)	(1)	(2)	(3)	(4)	(5)
	Health	Health	Health	Health	Health	Health	Health	Health	Health	Health
L.health	0.320 ***	0.347 ***	0.327 **	0.254 **	0.301 **	0.419 ***	0.515 ***	0.500 ***	0.325 ***	0.374 ***
	(3.03)	(2.68)	(2.40)	(2.40)	(2.25)	(3.08)	(3.04)	(2.89)	(3.29)	(3.07)
theil	1.091 ***	1.061 ***	0.973 ***	0.924 ***	0.900 ***	1.017 ***	1.051 ***	0.987 ***	0.921 ***	0.903 ***
	(8.00)	(8.90)	(7.69)	(6.55)	(5.68)	(8.53)	(8.34)	(7.32)	(8.62)	(7.08)
edu		0.002	0.001	0.014 *	0.015 **		0.004	0.002	0.020 ***	0.021 ***
		(0.40)	(0.15)	(1.78)	(1.99)		(0.75)	(0.48)	(2.80)	(2.73)
pdi			0.010	0.022	0.091			0.011	0.026	0.060
			(0.21)	(0.55)	(1.12)			(0.21)	(0.42)	(0.78)
dep				−0.025 *	−0.028 **				−0.032 ***	−0.034 ***
				(−1.65)	(−2.01)				(−2.76)	(−2.73)
gdp					−0.036					−0.022
					(−1.03)					(−0.65)
N	176	176	176	176	176	176	176	176	176	176
AR(1)-*p*	0.005	0.008	0.009	0.015	0.014	0.022	0.020	0.022	0.024	0.023
AR(2)-*p*	0.617	0.603	0.727	0.824	0.697	0.644	0.533	0.600	0.696	0.615
Sargan-*p*	0.217	0.173	0.128	0.077	0.146	0.217	0.173	0.128	0.077	0.146
Hansen-*p*	0.222	0.332	0.296	0.188	0.223	0.222	0.332	0.296	0.188	0.223

Note: z statistics in parentheses; * *p* < 0.1, ** *p* < 0.05, *** *p* < 0.01.

**Table 6:** Regression results of the fixed effect model and random effect model.

Variable	Fixed Effect Model					Random Effect Model				
	(1)	(2)	(3)	(4)	(5)	(1)	(2)	(3)	(4)	(5)
	Health	Health	Health	Health	Health	Health	Health	Health	Health	Health
theil	0.355 ***	0.353 ***	0.345 ***	0.404 ***	0.413 ***	0.487 ***	0.699 ***	0.763 ***	0.916 ***	1.102 ***
	(3.62)	(3.56)	(3.46)	(3.95)	(4.04)	(4.68)	(6.01)	(6.38)	(7.73)	(8.53)
edu		−0.000	−0.001	0.008 *	0.009 *		0.011 ***	0.015 ***	0.037 ***	0.042 ***
		(−0.21)	(−0.69)	(1.70)	(1.90)		(6.26)	(7.25)	(8.08)	(9.36)
pdi			0.021	0.022	0.065 *			−0.057 *	−0.041	−0.052
			(0.84)	(0.88)	(1.75)			(−1.88)	(−1.39)	(−1.17)
dep				−0.016 **	−0.019 **				−0.041 ***	−0.042 ***
				(−2.19)	(−2.48)				(−5.09)	(−5.09)
gdp					−0.023					0.003
					(−1.55)					(0.20)
cons	0.011	0.022	−0.017	−0.153	−0.173*	0.016	−0.316**	−0.254*	−0.564 ***	−0.688 ***
	(1.05)	(0.43)	(−0.24)	(−1.64)	(−1.84)	(0.08)	(−2.29)	(−1.75)	(−3.83)	(−4.98)
N	264	264	264	264	264	264	264	264	264	264
Within R^2^	0.0565	0.0567	0.0598	0.0802	0.0904	0.0565	0.0241	0.0175	0.0415	0.0359
Adj R^2^	0.9910	0.9909	0.9909	0.9911	0.9911	0.9910	0.9909	0.9909	0.9911	0.9911

Note: For the random effect model, the value in parentheses was the z statistic; for the fixed effect model, the value in parentheses was the t statistic; * *p* < 0.1, ** *p* < 0.05, *** *p* < 0.01.

**Table 7:** Regression results of two-way fixed effect model.

Variable	Two-Way Fixed Effect Model				
	(1)	(2)	(3)	(4)	(5)
	Health	Health	Health	Health	Health
theil	0.359 ***	0.355 ***	0.357 ***	0.462 ***	0.462 ***
	(3.60)	(3.52)	(3.61)	(4.59)	(4.58)
edu		−0.000	−0.004 *	0.012 **	0.012 **
		(−0.20)	(−1.84)	(2.39)	(2.37)
pdi			0.303 ***	0.421 ***	0.424 ***
			(3.18)	(4.28)	(4.25)
dep				−0.028 ***	−0.028 ***
				(−3.58)	(−3.55)
gdp					0.003
					(0.20)
Individual fixed Effect	yes	yes	yes	yes	yes
Time fixed Effect	yes	yes	yes	yes	yes
cons	0.009	0.020	−0.716 ***	−1.247 ***	−1.270 ***
	(0.37)	(0.34)	(−3.00)	(−4.52)	(−4.26)
N	264	264	264	264	264
Within R^2^	0.0570	0.0572	0.1000	0.1515	0.1517
Adj R^2^	0.9908	0.9907	0.9911	0.9916	0.9915

Note: t statistics in parentheses; * *p* < 0.1, ** *p* < 0.05, *** *p* < 0.01.

**Table 8:** A comprehensive summary of regression results.

Variable	One-Step System GMM Model	Two-Step System GMM Model	Fixed Effect Model	Two-Way Fixed Effect Model
	(1)	(2)	(3)	(4)
	Health	Health	Health	Health
L.health	0.301 **	0.374 ***		
	(2.25)	(3.07)		
theil	0.900 ***	0.903 ***	0.413 ***	0.462 ***
	(5.68)	(7.08)	(4.04)	(4.58)
edu	0.015 **	0.021 ***	0.009 *	0.012 **
	(1.99)	(2.73)	(1.90)	(2.37)
pdi	0.091	0.060	0.065 *	0.424 ***
	(1.12)	(0.78)	(1.75)	(4.25)
dep	−0.028 **	−0.034 ***	−0.019 **	−0.028 ***
	(−2.01)	(−2.73)	(−2.48)	(−3.55)
gdp	−0.036	−0.022	−0.023	0.003
	(−1.03)	(−0.65)	(−1.55)	(0.20)
Individual fixed Effect			yes	yes
Time fixed Effect			no	yes
N	176	176	264	264
Within R^2^			0.0904	0.1517
Adj R^2^			0.9911	0.9915
AR(1)-*p*	0.014	0.023		
AR(2)-*p*	0.697	0.615		
Sargan-*p*	0.146	0.146		
Hansen-*p*	0.223	0.223		

Note: For the system GMM model, the value in parentheses was the z statistic; for the fixed effect model and the two-way Fixed Effect Model, the value in parentheses was the t statistic; * *p* < 0.1, ** *p* < 0.05, *** *p* < 0.01.

## Data Availability

Not applicable.
